# Genome Sequences of Four Strains of Acinetobacter bereziniae Isolated from Human Milk Pumped with a Personal Breast Pump and Hand-Washed Milk Collection Supplies

**DOI:** 10.1128/MRA.00770-20

**Published:** 2020-10-29

**Authors:** Sarah M. Reyes, Emilienne Bolettieri, Dainelle Allen, Anthony G. Hay

**Affiliations:** a Division of Nutritional Sciences, Cornell University, Ithaca, New York, USA; b Department of Microbiology, Cornell University, Ithaca, New York, USA; Indiana University, Bloomington

## Abstract

Acinetobacter bereziniae, formerly Acinetobacter genomospecies 10, is an opportunistic pathogen possessing resistance to multiple antibiotics, and it has been reported to be responsible for hospital-associated infections in immunocompromised individuals. We report the draft genome sequences of four Acinetobacter bereziniae strains that were isolated from a single human milk sample collected with a personal breast pump and a hand-washed milk collection kit.

## ANNOUNCEMENT

Acinetobacter bereziniae, formerly known as Acinetobacter genomospecies 10, is a strictly aerobic, nonfermentative, nonmotile Gram-negative coccobacillus and a member of the class *Gammaproteobacteria* (1, 2). It is considered an emerging nosocomial pathogen (3), and several isolates of A. bereziniae have been reported to be responsible for health care-associated infections, including sepsis (1, 4–6). Additionally, isolates of A. bereziniae have been reported to be resistant to multiple antibiotics, including penicillins, carbapenems, and other β-lactams (1, 5, 7). Acinetobacter species are common in the environment (1) and have been detected as contaminants in human and animal milks (8–12). They are noted for their surface hydrophobicity, which contributes to their adherence to surfaces (13). Other species of Acinetobacter have been linked to nosocomial outbreaks in neonatal intensive care units; the sources of infection in some cases have been breast pumps and milk collection equipment (14–16). While Acinetobacter baumannii and Acinetobacter septicus have received significant attention in the literature (16–21), A. bereziniae has received little attention.

Four strains of A. bereziniae were isolated from human milk samples collected as part of the Milk in Life Conditions (MiLC) Trial (*n* = 52) (ClinicalTrials.gov registration number NCT03123874). MiLC was an in-home randomized, controlled, crossover trial to test the effects of women’s own pumping practices on the human milk microbiome. Participants provided written informed consent according to the study protocol approved by the Cornell University institutional review board (protocol 1608006566). These isolates were obtained from a single woman whose milk was pumped with her personal electric breast pump fitted with her own milk collection kit, which was washed by hand in the woman’s home kitchen. The woman’s infant was 4 months old and was reported to be healthy.

Milk samples were plated on Leeds Acinetobacter medium (Hardy Diagnostics, Santa Maria, CA) and were grown aerobically at 37°C for 18 h; colonies were purified by restreaking three times. DNA was isolated via freeze-thawing of cells and then was purified with glass milk (22). Sanger sequencing of the 16S rRNA gene was performed on four pure cultures (Table 1). Although they had 16S genes almost identical to each other (99.9%) and to those of the type strain A. bereziniae LMG1003 (average of 99.3% identity over 970 bp) (2, 23), ribosomal intergenic spacer analysis (RISA) demonstrated the presence of two distinct groups of isolates (24). All four were thus selected for whole-genome sequencing so that we could examine differences within and between RISA groups.

**TABLE 1:** Summary of whole-genome assemblies for the A. bereziniae strains in this report

Sample	Source^*a*^	GenBank accession no. for:		Length (bp)^*b*^	Read coverage (×)^*c*^	No. of contigs^*b*^	*N*_50_ (bp)	No. of predicted genes	GC content (%)	Completeness (%)	Contamination (%)	SRA accession no.
		16S rRNA sequence	Whole-genome sequence									
A005.1	Human milk	MT920178	JACDXR000000000	4,888,305	662	174	66,526	2,055	37.91	99	1.77	SRR12713660
A005.2	Human milk	MT920179	JABWPZ000000000	4,892,939	604	158	74,182	2,065	37.89	99	1.70	SRR12713659
A005.3	Human milk	MT920180	JABWPY000000000	4,902,916	303	187	81,363	2,068	37.89	99	1.72	SRR12713658
A005.4	Human milk	MT920181	JACDXQ000000000	4,875,542	353	318	31,353	2,093	38.06	99	1.70	SRR12713657

a All samples were sourced from a single human milk sample collected from a woman at home using her own personal electric breast pump and milk collection supplies, which were washed by hand in her home kitchen.

b The data provided for these parameters include all contigs that are greater than 0 bp long.

c Calculated as the total number of reads per genome length.

For whole-genome sequencing, sequence libraries were prepared using the Nextera XT Flex kit (Illumina, Inc., San Diego, CA), followed by sequencing on an Illumina NextSeq v2.18 system, resulting in paired-end reads (2 × 150 bp). Raw reads were uploaded to KBase (25), where adaptor sequences were trimmed using Trimmomatic v0.36 (26) and low-quality reads were removed using FastQC v0.11.5img (27). The genomes were assembled *de novo* with SPAdes v3.13.0 (28) and quality controlled using QUAST (29) and CheckM v1.0.18 (30). The genomes were annotated using RAST (31). Default parameters were used for all software unless otherwise stated.

The draft genomes ranged in size from 4,889,305 to 4,902,916 bp (Table 1). The numbers of contigs ranged from 158 to 318, and the *N*_50_ values ranged from 31,353 to 81,363 bp, with the total numbers of predicted genes ranging from 2,055 to 2,093. The GC contents were similar among the isolates and ranged from 37.89 to 38.06%.

Little is known about the impact of Acinetobacter bereziniae from human milk on neonates, but the presence of multiple antibiotic resistance determinants and a hemolysin in these strains, along with the status of A. bereziniae as an emerging pathogen (3), suggests that further analysis of this genus beyond strains of the widely characterized species *A. baumannii* and *A. septicus* is warranted (17–19).

### Data availability.

The sequenced genomes have been deposited in NCBI GenBank under BioProject PRJNA639858. The sequences can be accessed with BioSample and SRA accession numbers SAMN15283204 and SRR12713660 (A005.1), SAMN15283205 and SRR12713659 (A005.2), SAMN15283206 and SRR12713658 (A005.3), and SAMN15283207 and SRR12713657 (A005.4), respectively. Accession numbers for 16S rRNA sequences and whole-genome sequences are available in Table 1.
